# Editorial: Parathyroid atypical adenomas and carcinoma: An update on clinical management

**DOI:** 10.3389/fendo.2023.1163996

**Published:** 2023-02-24

**Authors:** Laura Gianotti, Filomena Cetani

**Affiliations:** ^1^ Division of Endocrinology, Department Internal Medicine, S.Croce & Carle Hopsital, Cuneo, Italy; ^2^ Unit of Endocrinology, University Hospital of Pisa, Pisa, Italy

**Keywords:** parathyroid carcinoma, atypical adenoma, long non-coding RNAs, CDC73 mutations, ectopic localization

Atypical parathyroid adenomas and parathyroid carcinomas are rare among parathyroid tumors that commonly are benign. They share some histological features namely trabecular growth pattern, fibrous bands, and cellular atypia but parathyroid carcinoma shows unequivocal features of neoplastic infiltration of adjacent tissues, lympho-vascular invasion, or perineural invasion (1, 2). Atypical parathyroid adenomas represent a challenge for the differential diagnosis with parathyroid carcinomas (2). Recently, the new classification of the 2022 WHO Classification of Parathyroid Tumors no longer endorses the use of “atypical parathyroid adenoma”. This entity has now been labeled as “atypical parathyroid tumor” to reflect a parathyroid neoplasm of uncertain malignant potential (3). The prognosis of patients with parathyroid carcinoma mainly depends on early detection and a radical surgical treatment. A strict and continued follow-up is mandatory to detect relapse or metastasis (1, 3). The diagnosis of parathyroid carcinoma is quite difficult due to the lack of reliable clinical diagnostic criteria, and in most cases is made postoperatively at histological examination (1). The clinical manifestations are primarily due to the excessive secretion of PTH by the tumor rather than spread to local or distant organs. Over the past few decades there has been no significant trend toward detecting the disease at an earlier stage, with no significant change in patient survival. No specific guidelines addressed to the clinical management of patients with parathyroid carcinomas are present in literature.

The Research Topic on Parathyroid Atypical Adenomas and Carcinoma has given attention to these aspects and underlines the need of more studies in parathyroid tumor research. Accordingly, the interesting application of the bibliometric analysis by a machine learning-based method in primary hyperparathyroidism (PHPT) proposed by Zhang et al. showed that the annual scientific publications on parathyroid tumors have scarcely changed during the last two decades. On the other hand, some novel aspects on the molecular profile and genetics of parathyroid tumours are emerging and new evidence from imaging studies or medical treatment and surgical techniques have also appeared in recent years. In the Research topic an extensive review by Marini et al. summarized the genetic and molecular pathways of parathyroid carcinogenesis. The Authors focused their attention on the germline heterozygote inactivating mutations of the *CDC73* tumor suppressor gene that accounts for about 50-75% of familial cases; over 75% of sporadic parathyroid carcinomas harbor biallelic somatic inactivation/loss of *CDC73* and one-third of them are germline (1). Other novel gene mutations, alterations of the PI3K/AKT/mTOR signaling pathway as well as modifications of microRNA expression profile and gene promoter methylation pattern have been detected in parathyroid carcinoma (4).

The role of Long non-coding RNAs (lncRNAs) as prognostic and diagnostic biomarkers in parathyroid cancer has also been explored by Morotti et al. suggesting that human parathyroid tumors are characterized by a different lncRNAs signature, with the lncRNA BC200/BCYRN1 representing a candidate biomarker for parathyroid carcinoma and its possible use in the post-operative setting for patient’s follow-up.

Clinical aspects and new therapeutical approaches of parathyroid carcinomas have been extensively revised by Zelano et al. that reported a life-threatening case of parathyroid carcinoma, associated with several systemic manifestations. Furthermore, some clinical challenge due to ectopic localization of parathyroid tumours has been raised, as in the paper by Validazeh et al. in whom the first case of parathyroid adenoma in the lung has been reported and successfully resected with video-assisted thoracoscopic surgery.

In the setting of parathyroid tumors, atypical parathyroid adenomas are considered a distinct clinical entity often associated with more severe form of the disease. Accordingly, Bhan et al. presented five cases of PHPT with osteitis fibrosa cystica, an uncommon manifestation of contemporary PHPT associated with larger parathyroid adenomas. Lastly, it is worth mentioning the contribute of Tzikos et al. on the evaluation of quality of life (QoL) in patients with PHPT. Through the Pasieka’s parathyroid assessment of symptoms Questionnaire (PAS-Q) and the validation of this tool in Greek speaking population, they applied PAS-Q - a self-administered questionnaire, which was developed in Canada specifically to assess the symptoms of PHPT and the impact on patients’ lives. PAS-Q may be considered a useful method to evaluate PHPT patients’ QoL and should be encouraged for dissemination in European and Eastern countries.

In conclusion, new aspects are emerging on the pathogenesis and clinical aspects of parathyroid tumors. More attention needs to be given to the parathyroid disease including molecular and genetic aspects, the disease expression, and the influence on the QoL of the patients. All these aspects appear to be very important to optimize the diagnostic workout and individualize the therapeutic strategy of the patients.

## Author contributions

LG and FC equally contributed to conceptualization and writing the manuscript. All authors contributed to the article and approved the submitted version.
